# Supplementary material for: Non-Association of Driver Alterations in PTEN with Differential Gene Expression and Gene Methylation in IDH1 Wildtype Glioblastomas
Source: Brain Sci. 2023 Jan 23;13(2):186. doi: 10.3390/brainsci13020186 (PMC9953940; doi:10.3390/brainsci13020186)
Supplement: Supplementary file 1 [file brainsci-13-00186-s001.zip › Supplementary Methods.pdf]

SUPPLEMENTARY METHODS

The steps that were followed on 'Materials and Methods' have been detailed below and numbered as sections for easy reference.

S1. Opening the dataset on Cbioportal.

S2. Creating a subset of cases of primary GB where profiles of CNAs, mutations and mRNA expression are concurrently available.

S3. Creating a subset of cases of primary GB where profiles of CNAs, mutations and DNA methylation data are concurrently available.

S4. Creating a subset of cases of primary GB where profiles of CNAs, mutations and protein expressions are concurrently available.

S5. Creating datasets of IDH1-W-GB from any subsets created in 2,3 and 4.

S6. Creating user-defined groups of patients with DA in selected genes.

S7. Creating user-defined groups of patients without DA in selected genes.

S8. Comparing user-defined groups.

S9. Creating user-defined groups of patients with combinations of DA in selected driver genes.

S10. Determining enriched Reactome pathways in a list of genes.

S11. Determining the enriched gene ontology (GO) terms in a list of genes.

S1. Opening the dataset of a study group on Cbioportal, following the example of the TCGA-Firehose Legacy Study group on Glioblastomas (TCGA-FL-SG on GB)

i. Open any internet browser and browse cbioportal.org.

ii. Create your user credentials using email id and then 'sign-in'.

iii. From the side menu on the homepage, select 'CNS/Brain'. This selection, opens a list of studies, one of which is 'Glioblastoma Multiforme (TCGA Firehose Legacy). Check the adjacent box.

iv. Click on the 'Explore Selected Study' link.

v. This will open a page that provides access to data from 619 samples obtained from 606 patients with GB.

vi. From the box named ' sample type', select ' Primary' leaving out ' Recurrences' .

vii. A page with data comprising of 606 samples from 606 primary GB patients opens.

S2. Creating a subset of cases of primary GBs where profiles of CNA, mutations and mRNA expressions are concurrently available following the example of the TCGA-FL-SG on GB

i. Within the box labeled 'genomic profile sample counts', check three boxes named 'Putative copy number alterations from GISTIC', 'mutations' and 'mRNA expression (RNA seq V2 RSEM).

ii. Click ' select samples' at the lower part of this box and click ' intersection' .

iii. This selection, opens a page comprising primary GB samples where results of all the above tests are concurrently available. (All the boxes, adjacent to names of these tests will show ' 100%' , thus confirming that every case in this group has all the required test results available.

S3. Creating a subset of cases of primary GBs where profiles of CNA, mutations and DNA methylations are concurrently available following the example of the TCGA-FL-SG on GB

i. Within the box labeled 'genomic profile sample counts', check three boxes named 'Putative copy number alterations from GISTIC', 'mutations' and 'methylation (Illumina HM450 platform.

ii. Click 'select samples' at the lower part of this box and click 'intersection'.

iii. This selection, opens a page comprising primary GB samples where results of copy number alterations, mutations, and DNA methylation (performed on the Illumina HM450 platform) are concurrently available.

S4. Creating a subset of cases of primary GBs where profiles of CNA, mutations and protein expressions are concurrently available following the example of the of the TCGA-FL-SG on GB

i. Within the box labeled 'genomic profile sample counts', check three boxes named 'Putative copy number alterations', 'mutations', 'protein expression (RPPA).

ii. Click 'select samples' at the lower part of this box and then click ' intersection'.

iii. This selection, opens a page comprising primary GB samples where results of CNAs, mutations and protein expressions are concurrently available.

S5. Creating a dataset of IDH1-W-GB from any of the subsets created in 2, 3 and 4

i. In any of the above pages of samples created through the steps shown in either 2,3, or 4, type ' IDH1: DRIVER', in the blank box adjacent to the blue colored ' Query' button and click ' Query' .

ii. This will open a new page of samples with DAs in IDH1.

iii. Click on the 'download' button. This step opens a new page.

iv. Click on the copy button adjacent to the phrase 'Unaltered samples: List of samples *without* alterations'. This step creates a copy link.

v. Go back to the initial page described in step i.

vi. Click on the 'Custom Selection' button. This step opens an adjacent text box.

vii. Click inside this text box and type 'Ctrl + v' on the keyboard to copy the link of cases created in step iv.

viii. Click on 'filter to listed samples'. This step will create a new page of samples without IDH1 driver gene alterations i.e., IDH1 wildtype samples.

ix. Click on the blue-colored 'Groups' button, under which click the button ' Create new group from selected samples*'.*

x. Thus, a user-defined core group of IDH1-W-GBs is created. This group gets listed under the ' Groups' button. Here, it can be identified by a name that the user assigns. All other user-defined groups created subsequently will be listed under the ' Groups' button.

S6. Creating user-defined groups of patients with driver gene alteration in selected genes. (Following the example of EGFR)

i. Click on the above-created core group of IDH1-W-GBs under the 'Groups' button. A page comprising these IDH1-W-GB opens up.

ii. Type 'EGFR: DRIVER', in the blank box adjacent to the blue-colored 'Query' button and click ' Query' .

iii. This opens a new page of samples with DAs in *EGFR*.

iv. Click the 'download' button. This click opens a new page.

v. Click on the 'copy' button adjacent to the phrase 'Altered samples: List of samples with alterations'. This click will create a copy link.

vi. Go back to the initial page of core IDH1-W-GB.

vii. Click on the ‘Custom Selection’ button. This opens an adjacent text box.
viii. Click inside this text box and then type ‘Ctrl + V’ to copy the link of cases created in step iv.
ix. Click on ‘filter to listed samples’. This click creates a new page of samples with DAs in *EGFR*.
x. Click on the blue-colored ‘Groups’ button, under which click the button ‘Create new group from selected samples’.
xi. A user-defined core group of samples with DAs in *EGFR* is created.

S7. Creating user-defined groups of patients without DAs in selected genes following the example of EGFR.

Follow the steps described above under 6, but in step iv, type ‘unaltered samples’ instead of ‘ altered samples’ to create a group comprising of samples without DAs in EGFR.

S8. Comparing user-defined groups

Groups to be compared can be selected from the list of user-defined groups created and listed under the ‘Groups’ button and then clicking on the ‘compare’ button. This click opens a new page with links related to characteristic features based on which the user wishes to compare the groups. These features which may be compared are ‘survival’ , ‘ clinical features’ , ‘ mutations’ , ‘ Copy-number’ , ‘ mRNA’ , ‘ protein’ and ‘ DNA methylation’ . Clicking on the links associated with these features opens individual pages showing the results of such comparisons. The results can be filtered down to only those statistically significant simply by checking the relevant box.

S9. Creating user-defined groups of patients with combinations of DAs in selected combinations of genes following the example of CDKN2A and PTEN

i. Under the ‘Groups’ button, select three groups. These are the ‘IDH1-W-GB’ group, *PTEN*+ group (samples with DAs in *PTEN*) and *CDKN2A+* group (samples with DAs in *CDKN2A)*. Then click on the ‘compare’ button. A new page opens *with a Venn diagram. From this* diagram, four groups of cases can be created i.e., *CDKN2A-PTEN-, CDKN2A-PTEN*+, CDKN2A+PTEN- and *CDKN2A+PTEN+*. These groups get listed under the ‘groups’ button. Any combination of genes with or without alterations can be used to create such user-defined groups.

S10. Creating user-defined groups of cases with different types of DAs among cases with DAs in PTEN

i. A user-defined group of cases consisting only of those with DAs in PTEN was created using a method like that described in section 6.

ii. The resulting page, which shows all cases with DAs in *PTEN*, also groups them according to the type of DA using a convenient color-coding scheme.

iii. This enables us to create further subgroups of cases, each with a particular type of DA in PTEN,

iv. These subgroups can be compared for DEM, DMGs and DEPs.

S11. Steps in DAVID for enriched gene ontology terms and pathways analyses

i) Open any internet browser and browse to https://david.ncifcrf.gov

ii) Go to 'Shortcut to David tools' and then click on 'Functional annotation'

iii) On the following page, click on 'upload'

iv) In the blank text box under 'Enter gene list', paste the list of genes of interest.

v) Under 'Select Identifier', select 'Official_Gene_Symbol'.

vi) Select species as 'Homo sapiens', and 'List type' as 'gene list'.

vii) Submit the list.

viii) In the following page click 'Gene Ontology'

ix) Under 'gene ontology', select the default selections of 'GOTERM_BP_DIRECT', 'GOTERM_CC_DIRECT' and 'GOTERM_MF_DIRECT for the gene ontology categories of 'biological processes', 'cellular components' and 'molecular functions' respectively. For each of these categories, click the 'chart' button.

x) Clicking on the 'chart' button will open the respective pages for each of the three categories, in which the significantly enriched GO terms can be found.

xi) Next, select pathways

xii) We used 'Reactome pathways'

xiii) Click 'chart' next to the 'Reactome pathways' to find the significantly enriched biological pathways under Reactome pathways.
