# Supplementary material for: Non-Association of Driver Alterations in PTEN with Differential Gene Expression and Gene Methylation in IDH1 Wildtype Glioblastomas
Source: Brain Sci. 2023 Jan 23;13(2):186. doi: 10.3390/brainsci13020186 (PMC9953940; doi:10.3390/brainsci13020186)
Supplement: Supplementary file 1 [file brainsci-13-00186-s001.zip › SUPPLEMENTARY TABLE LEGENDS.pdf]

SUPPLEMENTARY TABLE LEGENDS

1. **SUPPLEMENTARY TABLE S1A:** Lists of significantly upregulated and downregulated mRNAs in association with 12 common driver genes in IDH1-wildtype glioblastomas. UREG: Upregulated; DREG: Downregulated
2. **SUPPLEMENTARY TABLE S1B:** Lists of significantly hypermethylated and hypomethylated genes in association with 12 common driver genes in IDH1-wildtype glioblastomas. HYPERMETH: Hypermethylated; HYPOMETH: Hypomethylated
3. **SUPPLEMENTARY TABLE S1C:** List of significantly overexpressed and under expressed mRNAs in association with 12 common driver genes in IDH1-wildtype glioblastomas. OVEREXP: Overexpressed; UNDEREXP: Under expressed; SUPPLEMENTARY TABLE S2
4. **SUPPLEMENTARY TABLE S2**
   S2A: Bivariate analyses using Spearman rank correlation to determine if any significant correlation exists between the prevalence of DAs in the analyzed genes and the associated counts of differentially expressed mRNAs. UREG_MRNA: Upregulated mRNA; DREG_MRNA: Downregulated mRNA; DEM: Differentially expressed mRNAs
   S2B: Bivariate analyses using Spearman rank correlation to determine if any significant correlation exists between the prevalence of DAs in the analyzed genes and the associated counts of differentially methylated genes. HYPERMETH: Hypermethylated genes; HYPOMETH: Hypomethylated genes; DMG: Differentially methylated genes
5. **SUPPLEMENTARY TABLE S3A**. Association between DAs in PTEN with DAs in other 11 genes in group C1
6. **SUPPLEMENTARY TABLE S3B**. Association between DAs in PTEN with DAs in other 11 genes in group C2
7. **SUPPLEMENTARY TABLE S3C**. Association between DAs in PTEN with DAs in other 11 genes in group C3
8. **SUPPLEMENTARY TABLE S4**. Lists of differentially expressed mRNAs, differentially methylated genes and differentially expressed proteins in the C4 group where analyses were concurrently performed on differential gene expression, gene methylation and protein expression. UREG: Upregulated; DREG: Downregulated; HYPERMETH: Hypermethylated; HYPOMETH: Hypomethylated; OVEREXP: Overexpressed; UNDEREXP: Under expressed
9. **SUPPLEMENTARY TABLE S5:** The table shows the result of gene ontology analyses for DEMs in association with DAs in *CDKN2A*, *EGFR* and *TP53*.
10. **SUPPLEMENTARY TABLE S6:** The table shows the result of gene ontology analyses for DMG s in association with DAs in *CDKN2A*, *EGFR* and *TP53*.
11. **SUPPLEMENTARY TABLE S7:** The table shows the lists of DEMs that are common between those generated in association with DAs in CDKN2A, EGFR and TP53. Each column represents the two conditions that were compared separated by the ^ symbol (represents comparison). For example, the column A: CDKN2A-UREG ^ EGFR -UREG shows the lists of DEMs that are upregulated in association with DAs in CDKN2A

and upregulated in association with DAs in *EGFR*. UREG: Upregulated; DREG: Downregulated

12. **SUPPLEMENTARY TABLE S8:** The enriched molecular functions, biological processes, cellular components and Reactome pathways among lists of DEMs common between DAs in EGFR, CDKN2A and PTEN. The numbers in brackets indicate the numbers of genes in that list.
