# Supplementary material for: Non-Association of Driver Alterations in PTEN with Differential Gene Expression and Gene Methylation in IDH1 Wildtype Glioblastomas
Source: Brain Sci. 2023 Jan 23;13(2):186. doi: 10.3390/brainsci13020186 (PMC9953940; doi:10.3390/brainsci13020186)
Supplement: Supplementary file 1 [file brainsci-13-00186-s001.zip › Supplementary Table S2.pdf]

SUPPLEMENTARY TABLE S2

Supplementary Table S2A: Bivariate analyses using Spearman Rank correlation, show no significant correlation between the prevalence of DAs (C1) in the analyzed genes (n = 12) and their corresponding DEM counts (upregulated, downregulated, and total counts)

		**UREG_MRNA**	**DREG_MRNA**	**TOTAL_DEM**
**PREV_C1**	Spearman's rho Correlation coefficient	0.299	0.344	0.390
	p value	0.35	0.27	0.21

Supplementary Table S2B: Bivariate analyses using Spearman Rank correlation, show no significant correlation between the prevalence of DAs (C2) in the analyzed genes (n = 12) and their corresponding DMG counts (hypermethylated, hypomethylated, and total counts)

		**HYPERMETH**	**HYPOMETH**	**TOTAL_DMG**
**PREV_C2**	Spearman's rho Correlation coefficient	0.018	- 0.286	- 0.309
	p value	0.96	0.37	0.33

PREV_C1: Prevalence of DAs in C1 Upregulated, Downregulated and Total DMGs

PREV_C2: Prevalence of DAs in C2 Hypermethylated, Hypomethylated and total DMGs
