# Supplementary material for: Non-Association of Driver Alterations in PTEN with Differential Gene Expression and Gene Methylation in IDH1 Wildtype Glioblastomas
Source: Brain Sci. 2023 Jan 23;13(2):186. doi: 10.3390/brainsci13020186 (PMC9953940; doi:10.3390/brainsci13020186)
Supplement: Supplementary file 1 [file brainsci-13-00186-s001.zip › Supplementary Table S3A.pdf]

Supplementary Table S3A
Association Between DAs in *PTEN* with DAs in 11 Genes in C1 (N=129)

Genes	Odds Ratio	Confidence Interval	p value
CDK4	0.94	0.37-2.37	0.9
CDKN2A	0.85	0.41-1.73	0.85
EGFR	0.99	0.49-1.97	0.98
MDM2	1.2	0.41-4.69	0.79
MDM4	1.54	0.46-5.07	0.47
NF1	0.46	0.11-1.79	0.26
PDGFRA	0.43	0.14-1.29	0.14
PIK3CA	0.72	0.17-3.03	0.66
PIK3R1	0.73	0.12-4.13	0.72
RB1	4.34	1.28-14.71	0.0183
TP53	1.11	0.51-2.39	0.78
