# Supplementary material for: Non-Association of Driver Alterations in PTEN with Differential Gene Expression and Gene Methylation in IDH1 Wildtype Glioblastomas
Source: Brain Sci. 2023 Jan 23;13(2):186. doi: 10.3390/brainsci13020186 (PMC9953940; doi:10.3390/brainsci13020186)
Supplement: Supplementary file 1 [file brainsci-13-00186-s001.zip › Supplementary Table S3B.pdf]

**Supplementary Table S3B<br>Association Between DAs in *PTEN* with DAs in 11 Driver genes in group C2 (N=102)**			
**Names**	**Odds Ratios**	**Confidence Interval**	**p value**
CDK4	0.69	0.20-2.33	0.55
CDKN2A	0.67	0.28-1.59	0.36
EGFR	0.86	0.36-2.01	0.72
MDM2	0.8	0.19-3.24	0.75
MDM4	3.05	0.76-12.250.11	0.12
NF1	0.45	0.11-1.71	0.24
PDGFRA	0.32	0.06-1.53	0.15
PIK3CA	0.17	0.02-1.40	0.1
PIK3R1	0.5	0.13-1.91	0.31
RB1	2.35	0.55-10.09	0.24
TP53	1.02	0.37-2.83	0.95
