# Supplementary material for: Non-Association of Driver Alterations in PTEN with Differential Gene Expression and Gene Methylation in IDH1 Wildtype Glioblastomas
Source: Brain Sci. 2023 Jan 23;13(2):186. doi: 10.3390/brainsci13020186 (PMC9953940; doi:10.3390/brainsci13020186)
Supplement: Supplementary file 1 [file brainsci-13-00186-s001.zip › Supplementary Table S3C.pdf]

**Supplementary Table S3C<br>Association Between DAs IN *PTEN* WITH DAs IN 11 Driver genes Group C3 (N=132)**			
**Names**	**Odds Ratios**	**Confidence Interval**	**p value**
CDK4	0.39	0.12-1.2	0.11
CDKN2A	1.05	0.48-2.28	0.89
EGFR	0.69	0.33-1.43	0.32
MDM2	0.73	0.18-2.90	0.65
MDM4	1.44	0.44-4.70	0.54
NF1	0.39	0.10-1.42	0.15
PDGFRA	0.67	0.08-2.02	0.48
PIK3CA	0.41	0.48-2.02	0.27
PIK3R1	0.7	0.20-2.33	0.56
RB1	2.16	0.65-7.13	0.2
TP53	0.94	0.42-2.12	0.89
