# Supplementary material for: Non-Association of Driver Alterations in PTEN with Differential Gene Expression and Gene Methylation in IDH1 Wildtype Glioblastomas
Source: Brain Sci. 2023 Jan 23;13(2):186. doi: 10.3390/brainsci13020186 (PMC9953940; doi:10.3390/brainsci13020186)
Supplement: Supplementary file 1 [file brainsci-13-00186-s001.zip › Supplementary Table S5.pdf]

Supplementary Table S5

**Genes with DAs and the nature of differential mRNA expression**	**The gene ontology categories**	**The enriched gene ontology terms**
CDKN2A upregulated mRNAs (81)	Molecular functions	**1**. FBXO family protein binding **2**. Sphingosine N-acyltransferase activity **3**. Protein binding<br>**4**. Sulfuric ester hydrolase binding **5**. N-acyltransferase activity
	Biological processes	**1**. Epithelial cell proliferation **2.** Cilium assembly **3.** Hair follicle development<br>**4.** Regulation of centromere complex assembly **5**. Negative regulation of epithelial cell proliferation<br>**6**. Male gonad development **7**. Inactivation of MAPK pathway **8.** Motile cilium assembly
	Cellular components	**1**. Endoplasmic reticulum **2**. Endoplasmic reticulum membrane **3.** Endoplasmic reticulum lumen<br>**4**. Integral component of membrane
	Biological pathways	**1.** Sphingolipid metabolism **2.** Class B/2 (secretin family receptors) **3.** Metabolism of proteins **4.** Potassium channels **5.** Metabolism of lipids **6.** Defective binding of RB1 mutants to E2F1 (E2F2, E2F3) **7**. Aberrant regulation of mitotic G1/S regulation in cancer due to RB1 defects **8** Neuronal system **9.** Transmission across chemical synapses<br>**10**. Post translational protein modification
CDKN2A downregulated mRNAs (221)	Molecular functions	**1**. Chemorepellant activity **2.** Adenylate cyclase inhibiting G-protein coupled glutamate receptor activity<br>**3**.Voltage gated calcium channel activity **4**. Glutamate receptor activity **5**. G-protein coupled receptor activity involved in regulation of postsynaptic membrane potential **6**. Neurotransmitter receptor activity involved in regulation of postsynaptic cytosolic calcium ion concentration **7.** NAD transporter activity<br>**8.** Ubiquitin-protein transferase activity **9.** Sodium channel regulator activity **10**. RNA polymerase II transcription factor activity
	Biological processes	**1.** G protein coupled glutamate receptor signaling pathway **2.** Axon guidance<br>**3.** Adenylate cyclase inhibiting G-protein coupled glutamate receptor signaling pathway<br>**4**. Negative chemotaxis **5.** Protein autoubiquitination **6.** Regulation of ion transmembrane transport<br>**7.** Regulation of G-protein coupled receptor protein signaling pathway **8.** Cellular response to beta-amyloid<br>**9.** Embryo development **10.** Mitochondrial NAD transmembrane transport
	Cellular components	**1**. Glutaminergic synapse **2**. Voltage gated calcium channel complex **3.** Synapse **4.** Chromatin **5.** Anchoring junction<br>**6.** Astrocyte projection **7.** Transcription factor complex **8.** Neuron projection **9.** GABA-ergic response<br>**10.** Anchored component of pre-synaptic active zone membrane
	Biological pathways	**1.** GPCR downstream signaling **2**. Oncogene induced senescence **3.** Defective B3GALTL causes PpS

		**4.** Presynaptic depolarization and calcium channel opening **5.**O-glycosylation of TSR domain-containing proteins<br>**6.**Signaling by GPCR **7.** G1 Phase **8.** Cyclin D associated events in G1 **9.**Neuronal System<br>**10**.G alpha (q) signaling events
EGFR upregulated mRNAs (613)	Molecular functions	**1.** RNA polymerase II core promoter proximal region sequence-specific DNA binding 2. RNA polymerase II transcription factor activity, sequence-specific DNA binding **3.** metal ion binding **4.**Transcriptional activator activity, RNA polymerase II transcription regulatory region sequence-specific binding **5**.Transcriptional repressor activity, RNA polymerase II transcription regulatory region sequence-specific binding **6.** RNA polymerase II regulatory region sequence-specific DNA binding **7**. Transcription factor activity, sequence-specific DNA binding **8.** Sequence specific double stranded DNA binding **9**. Long chain fatty acid transporter activity **10.** Catalytic activity
	Biological processes	**1.** Regulation of transcription from RNA polymerase II promoter **2**. Regulation of transcription, DNA templated<br>**3.** Negative regulation of transcription from RNA polymerase II promoter **4**. Positive regulation of transcription from RNA polymerase II promoter **5.** Response to interferon beta **6.** Negative regulation of vascular permeability<br>**7.** Lacrimal gland development **8.** Neuron fate specification **9.** Ureteric bud development **10.** Positive development of osteoblast differentiation
	Cellular components	**1.** Nucleus **2.** Nucleoplasm **3.** Chromatin **4.** Cell junction **5.** Cell periphery **6.** Apical cell membrane **7.** Schimdt-Lanterman incisure **8**. Transcription factor complex **9.** Peroxisomal matrix **10.** Recycling endosome membrane
	Biological pathways	**1**. Generic transcription pathway **2.** RNA polymerase II transcription **3.** Gene expression (transcription)<br>**4.** Lysine catabolism **5**. Interferon alpha/beta signaling **6.** Interferon gamma signaling 7. Creatine metabolism<br>**8.** Pyroptosis **9.** Diseases of metabolism **10**. Sodium/calcium exchangers
EGFR downregulated mRNAs (664)	Molecular functions	**1.** Chemorepellant activity **2.** Growth factor activity **3.** Metalloendopeptidase activity **4**. Receptor agonist activity<br>**5**.Semaphorin receptor activity **6.** Guanyl nucleotide exchange factor activity **7.** BMP receptor binding<br>**8.** Transmembrane receptor protein tyrosine kinase activator activity **9.** Protein binding and bridging **10.** Glucosyl ceramidase activity
	Biological processes	**1.**Negative chemotaxis **2.** Negative regulation of BMP signaling **3.** Epithelial cell proliferation<br>**4.** Wnt signaling pathway **5.** Negative regulation of epithelial cell proliferation **6**. Axon extension involved in axon guidance **7.** Wnt signaling pathway involved in somitogenesis **8.** Positive regulation of epithelial cell proliferation **9.** Collagen fibril organization **10.** Negative regulation of Wnt signaling pathway
	Cellular components	**1**. intracellular membrane-bounded organelle **2**. Late endosome **3.** Golgi apparatus **4.** Plasma membrane<br>**5**. Cytosol **6.** Perinuclear region of cytoplasm **7.** Anchoring junction **8**. Axon **9.** Extracellular matrix **10.** Post synaptic density

	Biological pathways	**1.** Membrane trafficking **2.** Clathrin mediated endocytosis **3.** Cargo recognition for Clathrin mediated endocytosis<br>4.Vesicle mediated transport **5.** Collagen formation **6**. Collagen biosynthesis by modifying enzymes **7**. Signaling by nuclear receptor **8.** Retrograde transport at the trans-Golgi network **9.** Transport of small molecules<br>**10.** Negative regulation of TCF-dependent signaling by WNT ligand antagonists
TP53 upregulated mRNAs (1000)	Molecular functions	**1.** RNA binding **2.** Protein binding **3**. DNA binding **4.** Chromatin binding **5.** ATP binding **6.** ATPase activity<br>**7.** mRNA binding **8.** DNA helicase activity **9.** Single stranded DNA binding **10.** Nucleic acid binding
	Biological processes	**1.** Cell division **2.** Cell Cycle **3**. DNA repair **4**. Chromatin organization **5**. Cellular response to DNA damage stimulus<br>**6.** Positive regulation of transcription, DNA templated **7**. Mitotic cell cycle **8.** mRNA Splicing via spliceosome<br>**9.** Chromatin remodeling **10.** mRNA processing
	Cellular components	**1.** Nucleoplasm **2.** Nucleus **3**. Chromosome **4.** Cytosol **5**. Centrosome **6**. Nuclear speck **7**. Nucleolar<br>**8**. Spindle pole **9**. Cytoplasm **10**. Kinetochore
	Biological pathways	**1.** Cell cycle **2**. Cell cycle mitotic **3.** Gene expression (transcription) **4.** Metabolism of RNA **5**. M phase<br>**6.** Transcriptional regulation by TP53 **7.** Cell cycle checkpoints **8.** Mitotic prometaphase<br>**9**. Processing of capped intron containing Pre-MRNA **10.** RNA polymerase II transcription
TP53 downregulated mRNAs (285)	Molecular functions	**1.** Protein binding **2.** Iron.sulfur cluster binding **3.** Oxidoreductase activity **4.** NADH dehydrogenase ubiquinone activity **5.** Ubiquitin protein transferase activity **6.** Integrin binding **7.** Ion channel binding **8.** Methyltransferase activity **9.** Glucoronosyl transferase activity **10.** Misfolded protein binding
	Biological processes	**1.** protein polyubiquitination **2.** Mitochondrial electron transport, NADH to ubiquinone<br>**3.** regulation of high voltage-gated calcium channel activity **4.** Intrinsic apoptotic signaling pathway in response to DNA damage by p53 class mediator **5**. Lysosome localization **6.** Regulation of endosome size **7**. Mitochondrial respiratory chain complex I assembly **8.** Regulation of cell cycle **9.** Wound healing **10.** Positive regulation of endothelial cell migration
	Cellular components	**1.** Mitochondrion 2. Endoplasmic reticular membrane **3**. Mitochondrial inner membrane 4. Endoplasmic reticulum<br>**5.** Endosome membrane **6.** Integral component of membrane **7**. Golgi membrane **8.** Golgi apparatus **9.** Lysosomal membrane **9**. Ubiquitin ligase complex **10.** Mitochondrial respiratory chain complex 1
	Biological pathways	**1.** Metabolism **2**. Respiratory electron transport **3.** Complex I biogenesis **4.** Respiratory electron transport, ATP synthesis by chemiosmotic coupling, and heat production by uncoupling proteins. **5.**The citric acid (TCA) cycle and respiratory electron transport **6.** Diseases of signal transduction by growth factor receptors and second messengers **7.** Metabolism of proteins **8.** Mitochondrial iron-sulfur cluster biogenesis **9.** TNFR2 non-canonical NF-kB pathway **10.** Neddylation

Supplementary Table S5. The table shows the result of gene ontology analyses for *CDKN2A*, *EGFR* and *TP53*. The numbers in parenthesis represent the numbers of differentially expressed mRNAs. The gene ontology terms have been obtained using the DAVID functional annotation and pathway enrichment tools. The terms have been arranged in descending order of significance according to the p values. For pathway enrichment, the enriched pathways in "Reactome Pathways" have been shown. A maximum of 10 significantly enriched terms have been shown for each set of mRNAs when the number of enriched terms exceeds 10. The lists of differentially methylated mRNAs that were submitted for the analyses are available in Supplementary table S1A.
