# Supplementary material for: Non-Association of Driver Alterations in PTEN with Differential Gene Expression and Gene Methylation in IDH1 Wildtype Glioblastomas
Source: Brain Sci. 2023 Jan 23;13(2):186. doi: 10.3390/brainsci13020186 (PMC9953940; doi:10.3390/brainsci13020186)
Supplement: Supplementary file 1 [file brainsci-13-00186-s001.zip › Supplementary Table S6.pdf]

Supplementary Table S6

**Genes with DAs and the nature of differential gene methylation**	**The gene ontology categories**	**The enriched gene ontology terms**
CDKN2A hypermethylated genes (1)	Molecular functions	None
	Biological processes	None
	Cellular components	None
	Biological pathways	None
CDKN2A hypomethylated genes (2)	Molecular functions	None
	Biological processes	None
	Cellular components	None
	Biological pathways	None
EGFR hypermethylated genes (34)	Molecular functions	**1**. Zinc ion binding **2.** Signaling receptor activity **3.** Peptide hormone binding **4.** Receptor tyrosine kinase binding
	Biological processes	**1.** Positive regulation of cell division **2.** Cardiac muscle hypertrophy<br>**3.** Smooth muscle hypertrophy **4.** Cell-cell adhesion **5.** Positive regulation of nitric oxide synthetase activity **6.** Phospholipid transport<br>**7**. phosphatidylinositol 3-kinase signaling **8.** phosphatidylinositol 3-kinase signaling **9.** Neural crest migration **10.** Phosphatidylinositol mediated signaling
	Cellular components	**1.** Plasma membrane **2**. External side of plasma membrane **3.** Anchoring function **4.** Extracellular exosome **5**. Integral component of plasma membrane **6.** Perinuclear region of cytoplasm
	Biological pathways	**1**. Signal transduction
EGFR hypomethylated genes (9)	Molecular functions	None
	Biological processes	**1.** Protein localization to membrane **2.** Aging

	Cellular components	None
	Biological pathways*	None
TP53 hypermethylated genes (2)	Molecular functions	None
	Biological processes	None
	Cellular components	None
	Biological pathways	None
TP53 hypomethylated genes (22)	Molecular functions	**1.**PDZ domain binding
	Biological processes	**1**. Regulation of synaptic vesicle exocytosis **2.** Neurotransmitter receptor localization of postsynaptic specialization membrane **3.** Central nervous system development
	Cellular components	**1.** Plasma membrane **2.** Glutametargic synapse **3.** Dendrite **4.** Anchoring function **5**. Schaffer collateral **6.**Transport vesicle **7.** Sarcolemma **8.** Anchored component of membrane
	Biological pathways	**1.** Glutamate binding, activation of AMPA receptors and synaptic plasticity **2.** Trafficking of AMPA receptors

Supplementary Table S6. The table shows the result of gene ontology analyses for CDKN2A, EGFR and TP53. The numbers in parenthesis represent the numbers of differentially methylated genes. The gene ontology terms have been obtained using the DAVID functional annotation and pathway enrichment tool. The terms have been arranged in descending order of significance according to the p values. For pathway enrichment the enriched pathway sin "Reactome Pathways" was used. A maximum of 10 enriched terms have been shown for each set of differentially methylated when the number of enriched terms exceeds 10. The lists of differentially methylated genes are available in Supplementary table S1B.
