# Supplementary material for: Non-Association of Driver Alterations in PTEN with Differential Gene Expression and Gene Methylation in IDH1 Wildtype Glioblastomas
Source: Brain Sci. 2023 Jan 23;13(2):186. doi: 10.3390/brainsci13020186 (PMC9953940; doi:10.3390/brainsci13020186)
Supplement: Supplementary file 1 [file brainsci-13-00186-s001.zip › Supplementary Table S8.pdf]

Supplementary Table S8

Genes with DAs and the nature of differential mRNA expression	The gene ontology categories	The enriched gene ontology terms
mRNAs upregulated both in association with *EGFR* and *CDKN2A* *(12)*	Molecular functions	None
	Biological processes	1. Hair follicle development 2. Regulation of G-protein coupled receptor signaling pathway 3. Positive regulation of fibroblast proliferation
	Cellular components	1. Plasma membrane 2. Mitochondrial outer membrane
	Biological pathways	1. Cooperation of PDCL and TRiC/CCT in G-protein binding 2.Regulation of insulin secretion 3. Chaperonin mediated protein folding 4.Protein folding 5. Potassium channels 6. Integration of energy metabolism
mRNAs downregulated both in association with *EGFR* and *CDKN2A* *(25)*	Molecular functions	1. Chemorepellant activity
	Biological processes	1.Axon guidance 2. Negative chemotaxis
	Cellular components	1. Endoplasmic reticulum membrane 2.Pre-synaptic active zone 3.Glutametrgic response 4. Extracellular exosome
	Biological pathways	1. Transmission across chemical synapses
mRNAs upregulated in association with *CDKN2A* and downregulated in association with *TP53* *(14)*	Molecular functions	None
	Biological processes	1.Response to hypoxia
	Cellular components	1.None
	Biological pathways	1. Metabolism of proteins
mRNAs downregulated in association with *CDKN2A* and upregulated in association with *TP53* *(55)*	Molecular functions	**1.** RNA binding
	Biological processes	1.rRNA processing 2. Positive regulation of gene expression 3. Positive regulation of apoptotic processes 4. Regulation of osmotic stress 5.Positive regulation of glial cell proliferation 6. Negative regulation of transcription, DNA templated 7. Apoptotic process 8. Heterochromatin assembly 9. RNA splicing
	Cellular components	1. Nucleoplasm 2. Astrocyte projection 3. Chromatin
	Biological pathways	1. Oncogene induced senescence
mRNAs upregulated in association with *EGFR* and downregulated in association with *TP53* *(39)*	Molecular functions	1. Integrin binding
	Biological processes	1.Creatine biosynthetic process 2. Creatine metabolic process 3.Positive regulation of MAP kinase activity 4. Positive regulation of protein kinase B signaling 5. Supramolecular fiber organization 6. Ubiquinone biosynthetic process 7. Regulation of peptidyl-tyrosine phosphorylation 8. Intrinsic apoptotic pathway by p53 class mediator 9. Positive regulation of phosphorylation 10. Positive regulation of cell proliferation
	Cellular components	1.Mitochondrion 2.Integral component of membrane 3.Plasma membrane 4.Cell periphery
	Biological pathways	1. Creatine metabolism
mRNAs downregulated in association with *EGFR* and	Molecular functions	1. RNA binding 2. Phosphotyrosine binding 3. ATP binding 4. Manganese ion binding 5. Histone demethylase activity (H3-K36 specific) 6. Protein binding bridging 7. Ubiquitin protein transferase activity 8. Histone demethylase activity

upregulated in association with *TP53* *(84)*	Biological processes	1.rRNA processing 2. Golgi disassembly 3. Neural crest formation 4. Spermatogenesis 5. Histone H3-K36 demethylation 6. Wnt signaling pathway 7. Protein phosphorylation 8. Proteasome mediated ubiquitin dependent protein catabolic process 9. Insulin like growth factor receptor signaling pathway
	Cellular components	1. Nucleoplasm 2. Cytosol 3. Intracellular membrane bound organelle 4. Cytoplasm 5. Nucleus 6. Golgi apparatus 7. Nucleolus
	Biological pathways	1. Membrane trafficking 2. Vesicle mediated transport 3. Regulation of TP53 activity 4. Aspargine N-linked glycosylation 5. Transport to the golgi and subsequent modification 6. Intra-golgi and retrograde Golgi to ER traffic 7. Regulation of TP53 activity through phosphorylation 8. Signaling by Receptor tyrosine kinase 9. Post-translational modification

Supplementary Table S8: The table shows the result of gene ontology analyses for lists of genes that are commonly upregulated or downregulated in association with *CDKN2A*, *EGFR* and *TP53*. The numbers in parenthesis represent the numbers of differentially expressed mRNAs. The gene ontology terms have been obtained using the DAVID functional annotation and pathway enrichment tools. The terms have been arranged in descending order of significance according to the p values. For pathway enrichment, the enriched pathways in “Reactome Pathways” have been shown. The lists of differentially methylated mRNAs that were submitted for the analyses are available in Supplementary Table S7
